# Interacting Proteins on Human Spermatozoa: Adaptive Evolution of the Binding of Semenogelin I to EPPIN 

**DOI:** 10.1371/journal.pone.0082014

**Published:** 2013-12-02

**Authors:** Erick J. R. Silva, Katherine G. Hamil, Michael G. O’Rand

**Affiliations:** Department of Cell Biology & Physiology, University of North Carolina at Chapel Hill, Chapel Hill, North Carolina, United States of America; Inserm, France

## Abstract

Semenogelin I (SEMG1) is found in human semen coagulum and on the surface of spermatozoa bound to EPPIN. The physiological significance of the SEMG1/EPPIN interaction on the surface of spermatozoa is its capacity to modulate sperm progressive motility. The present study investigates the hypothesis that the interacting surface of SEMG1 and EPPIN co-evolved within the Hominoidea time scale, as a result of adaptive pressures applied by their roles in sperm protection and reproductive fitness. Our results indicate that some amino acid residues of SEMG1 and EPPIN possess a remarkable deficiency of variation among hominoid primates. We observe a distinct residue change unique to humans within the EPPIN sequence containing a SEMG1 interacting surface, namely His92. In addition, Bayes Empirical Bayes analysis for positive selection indicates that the SEMG1 Cys239 residue underwent positive selection in humans, probably as a consequence of its role in increasing the binding affinity of these interacting proteins. We confirm the critical role of Cys239 residue for SEMG1 binding to EPPIN and inhibition of sperm motility by showing that recombinant SEMG1 mutants in which Cys239 residue was changed to glycine, aspartic acid, histidine, serine or arginine have reduced capacity to interact to EPPIN and to inhibit human sperm motility in vitro. In conclusion, our results indicate that EPPIN and SEMG1 rapidly co-evolved in primates due to their critical role in the modulation of sperm motility in the semen coagulum, providing unique insights into the molecular co-evolution of sperm surface interacting proteins.

## Introduction

In primates, semen is a complex biological fluid containing spermatozoa bathed by the seminal plasma, which nurtures spermatozoa while providing the appropriate conditions for the last steps of post-testicular sperm maturation, and protecting them from pathogenic threats during their journey towards fertilization in the female reproductive tract [1]. The seminal vesicle-secreted protein semenogelin I (SEMG1) is the major component of the seminal plasma [2]. Immediately after ejaculation, human semen undergoes a coagulation process forming a gelatinous mass that contains SEMG1 as its structural element [3]. SEMG1 is found in the semen coagulum and on the surface of spermatozoa bound to EPPIN, a member of the whey-acidic protein (WAP)-type four-disulfide core (WFDC) family [4,5]. After ejaculation, the hydrolysis of SEMG1 by activated prostate-specific antigen (PSA), a serine protease, results in the liquefaction of the semen coagulum allowing spermatozoa to acquire progressive motility [2,6]. 

The physiological significance of the SEMG1/EPPIN interaction on the surface of spermatozoa is its capacity to modulate sperm progressive motility upon ejaculation in a finely time-regulated manner [5,7]. In parallel, EPPIN inhibits the digestion of SEMG1 by PSA, which results in the modulation of semen liquefaction, further prolonging the inhibitory effects of SEMG1 on sperm motility [8]. This effect is thought to be important for reproduction because it allows spermatozoa to achieve their full fertilizing capacity at the appropriate moment. Men whose semen coagulum fails to liquefy spontaneously have spermatozoa with poor motility and are infertile [9]. Additionally, EPPIN and some SEMG1-proteolytic peptides derived by PSA cleavage have strong antibacterial activity in vitro and may protect the integrity of spermatozoa against microorganisms present in the vaginal environment [10-13]. The critical roles of SEMG1 and EPPIN in reproduction are highlighted by the fact that male monkeys immunized with human recombinant EPPIN that developed high titers of anti-EPPIN antibodies lacked semen coagulum upon ejaculation and became reversibly infertile [14]. Subsequent studies demonstrated that anti-EPPIN antibodies isolated from immunized monkeys blocked the SEMG1 binding site on EPPIN’s C-terminal region and mimicked SEMG1 binding by inhibiting progressive motility of human spermatozoa [15,16]. Because of these observations, the SEMG1 binding interface on EPPIN has been acknowledged as a potential target for male contraception [5].

The WFDC locus on human chromosome 20q13, containing the *SEMG1* (in the centromeric cluster) and *EPPIN* (in the telomeric cluster) genes [17,18], has undergone strong adaptive pressure [19] and the *SEMG1* gene in particular has undergone rapid adaptive evolution [18-21], suggesting positive selection driven by their functions in natural immunity and reproductive success [19]. Genes for serine protease inhibitors within the WFDC locus may have been progenitors to the *SEMG* genes [17,22]. In previous studies on the interaction of SEMG1 with EPPIN on the sperm surface, we demonstrated that EPPIN’s Cys102, Tyr107, and Phe117 were necessary for SEMG1 binding [23] and that SEMG1’s Cys239 was required for binding to EPPIN with subsequent inhibition of sperm motility [24,25]. The SEMG1 amino acid residue Cys239 has been shown to be under positive selection [18]. Consequently we have asked whether EPPIN and SEMG1 have undergone co-adaptive evolution into a receptor ligand relationship that provides protection for spermatozoa and regulates the acquisition of progressive sperm motility prior to capacitation. Here we demonstrate that the Hominoidea underwent a distinct change in specific EPPIN and SEMG1 amino acid residues that appears to be a directional selection throughout the Hominidae (human and great apes) and Hylobatidae (gibbon) Families, resulting in a high affinity binding site between EPPIN and SEMG1 and a functional gain regarding its ability to inhibit sperm motility.

## Results

### Phylogenetic Analysis of EPPIN and SEMG1

**Figure 1:**
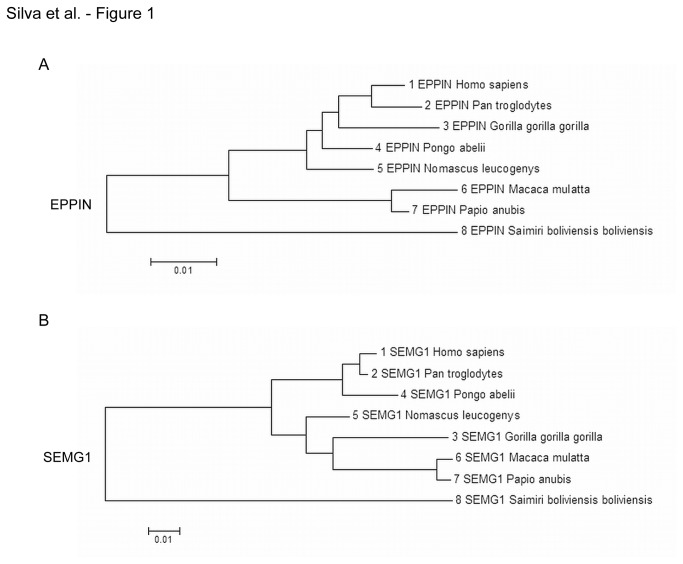
Molecular phylogenetic analysis for EPPIN and *SEMG1* within the primate lineage. EPPIN (**A**) and *SEMG1* (**B**) trees were drawn to scale with branch lengths measured in the number of substitutions per site. GenBank accession number for each DNA sequence used for EPPIN and SEMG1 analyses are shown in Table 6.

The Catarrhini (old world monkeys, great apes, gibbons, and humans) emerged 43.5 million years ago (MYA) from a common ancestor and subsequently diverged into the Hominoidea and Cercopithecoidea 31.6 MYA [26]. The Homininae emerged 8.3 MYA and underwent rapid speciation into three genera (*Gorilla, Pan, Homo*); the *Gorilla* branching rapidly away from the *Homo-Pan* genera [26]. We examined the molecular phylogeny of *EPPIN* and *SEMG1* within the Hominoidea and of *Macaca mulatta* and *Papio anubis* within the Cercopithecoidea and found 2.2 more substitutions per site in the *SEMG1* lineage (Figure **1A, B**). The Maximum Likelihood method gave the same tree for *EPPIN* (Figure **1A**) as all other methods and conformed to the expected evolutionary tree for primates [26]. However, using the same methodology, all the methods gave an unexpected tree for the *SEMG1* sequence (Figure **1B**) showing the *Gorilla* branching from the common ancestor of the Cercopithecoidea rather than the common ancestor of the Homininae. A test of the homogeneity of substitution patterns between sequences revealed that the *Gorilla EPPIN* sequence exhibits heterogeneity with *Homo*, *Pan* and *Pongo* (Table **1**, asterisks in row 1 vs. column 3, row 2 vs. column 3, and row 3 vs. column 4), whereas the *Gorilla* SEMG1 sequence exhibits heterogeneity with *Macaca mulatta* (Table **2**, asterisks in row 3 vs. column 6) and, although not statistically significant, a trend towards heterogeneity with *Homo sapiens* (Table **2**, trend symbol in row 1 vs. column 3, row 3 vs. column 6). Although there was an overabundance of non-synonymous substitutions in several codons in each lineage, only *SEMG1* showed positively selected codons that were statistically significant (Table **S1** and Table **S2**).

**Table 1:** Test of the homogeneity of substitution patterns between sequences for *EPPIN*.

**Species**	**1**	**2**	**3**	**4**	**5**	**6**	**7**	**8**
1 *Homo sapiens*	-	**0.000**	0.005*	0.002*	**0.000**	**0.000**	**0.000**	**0.000**
2 *Pan troglodytes*	*1.000*	-	0.005*	**0.000**	**0.000**	**0.000**	**0.000**	**0.000**
3 *Gorilla gorilla gorilla*	*0.314*	*0.320*	-	0.017*	**0.000**	**0.000**	**0.000**	**0.000**
4 *Pongo abelii*	*0.442*	*1.000*	*0.172*	-	**0.000**	0.015*	0.010*	**0.000**
5 *Nomascus leucogenys*	*1.000*	*1.000*	*1.000*	*1.000*	-	**0.000**	**0.000**	**0.000**
6 *Macaca mulatta*	*1.000*	*1.000*	*1.000*	*0.338*	*1.000*	-	**0.000**	**0.000**
7 *Papio anubis*	*1.000*	*1.000*	*1.000*	*0.324*	*1.000*	*1.000*	-	**0.000**
8 *Saimiri boliviensis boliviensis*	*1.000*	*1.000*	*1.000*	*1.000*	*1.000*	*1.000*	*1.000*	-

MEGA molecular evolutionary genetic analysis software package (version 5.1) [36] used a Monte Carlo test (500 replicates) to estimate the *p*-values [38], which are shown above the diagonal (bold). * *p*-values < 0.05 were considered significant. Estimates of the disparity index per site are shown for each sequence pair below the diagonal (italic). There were a total of 402 positions in the final dataset. GenBank accession number for each DNA sequence used for EPPIN and SEMG1 analyses are shown in Table **6**.

**Table 2:** Test of the homogeneity of substitution patterns between sequences for *SEMG1*.

**Species**	**1**	**2**	**3**	**4**	**5**	**6**	**7**	**8**
1 *Homo sapiens*	-	**0.000**	**0.051#**	0.011*	**0.000**	**0.000**	**0.000**	**0.000**
2 *Pan troglodytes*	*1.000*	-	**0.075**	0.011*	**0.000**	**0.000**	**0.000**	**0.000**
3 *Gorilla gorilla gorilla*	*0.192*	*0.102*	-	**0.192**	**0.141**	0.019*	**0.000**	**0.123**
4 *Pongo abelii*	*0.234*	*0.226*	*0.022*	-	**0.000**	**0.000**	0.039*	**0.000**
5 *Nomascus leucogenys*	*1.000*	*1.000*	*0.024*	*1.000*	-	**0.000**	**0.000**	**0.000**
6 *Macaca mulatta*	*1.000*	*1.000*	*0.280*	*1.000*	*1.000*	-	**0.000**	**0.000**
7 *Papio anubis*	*1.000*	*1.000*	*1.000*	*0.202*	*1.000*	*1.000*	-	**0.000**
8 *Saimiri boliviensis boliviensis*	*1.000*	*1.000*	*0.174*	*1.000*	*1.000*	*1.000*	*1.000*	-

MEGA molecular evolutionary genetic analysis software package (version 5.1) [36] used a Monte Carlo test (500 replicates) to estimate the *p*-values [38], which are shown above the diagonal (bold). * *p*-values < 0.05 were considered significant. Estimates of the disparity index per site are shown for each sequence pair below the diagonal (italic). There were a total of 738 positions in the final dataset. # indicates a trend towards heterogeneity, although it was not significant. GenBank accession number for each DNA sequence used for EPPIN and SEMG1 analyses are shown in Table **6**.

We found no evidence of positive selection in any site within the primate phylogenetic tree of *EPPIN* (Table **S1**). On the other hand, detailed examination of the full-length EPPIN sequence revealed that when the Hominoidea and Cercopithecoidea Superfamilies emerged from the Catarrhini 31.6 MYA the Hominoidea underwent a distinct change in seven amino acid residues (Lys35, Lys50, Asp51, Gln55, Lys81, Tyr89, Leu91, Met104, and Lys120) that appears to be a directional selection throughout the Hominidae and Hylobatidae Families (Table **3**), separating them from the Papionini. An examination of the EPPIN sequence within the SEMG1 binding site in the Kunitz domain [23] revealed only two residue changes that were different between *Homo* and all the other Hominoidea, namely His92 and Asp99. Only His92 projects into the binding pocket [23] and would represent a significant change from Arg92 in charge and size.

**Table 3:** Amino acid residue changes in the full-length EPPIN sequence.

**EPPIN**	**Species**						
Residues	**7**	**6**	**5**	**4**	**3**	**2**	**1**
(position #)			**^**				
8	S	S	S	***N***	S	S	S
14	I	I	I	***V***	***V***	***V***	***V***
17	V	A	A	A	***V***	A	A
35	T	T	***K***	***K***	***K***	***K***	***K***
43	K	K	K	K	***Q***	***Q***	***Q***
50	R	R	***K***	***K***	***K***	***K***	***K***
51	H	H	***D***	***D***	***D***	***D***	***D***
55	P	P	***Q***	***Q***	***Q***	***Q***	***Q***
78	E	E	E	E	E	***K***	E
81	N	N	***K***	***K***	***K***	***K***	***K***
89	F	F	***Y***	***Y***	***Y***	***Y***	***Y***
91	I	I	***L***	***L***	***L***	***L***	***L***
92	R	R	R	R	R	R	***H***
99	N	N	N	N	N	N	***D***
101	T	T	T	T	***S***	T	T
104	T	T	***M***	***M***	***M***	***M***	***M***
107	Y	H	Y	Y	Y	Y	Y
111	Q	Q	***P***	Q	Q	Q	Q
120	E	E	***K***	***K***	***K***	***K***	***K***
128	K	K	K	K	***R***	K	K
129	N	N	N	***K***	N	N	N

N-terminal WAP domain and C-terminal Kunitz domain are present within positions 1-74 and 75-133 of EPPIN.

^ indicates the divergence of the Catarrhini into the Hominoidea (species 1-5) and Cercopithecoidea (species 6 and 7), 31.6 MYA; Changes after the split are shown in bold/italics. Residues 101 (Serine) and 128 (Arginine) in the *Gorilla* sequence represent changes unique to *Gorilla*. Residues 92 (Histidine) and 99 (Aspartic acid) in the *Homo sapiens* sequence represent changes unique to *Homo sapiens*. Species: 1, *Homo sapiens*; 2, *Pan troglodytes*; 3, *Gorilla gorilla*; 4, *Pongo abelii*; 5, *Nomascus leucogenys*; 6, *Macaca mulatta*; 7, *Papio anubis*.

**Figure 2:**
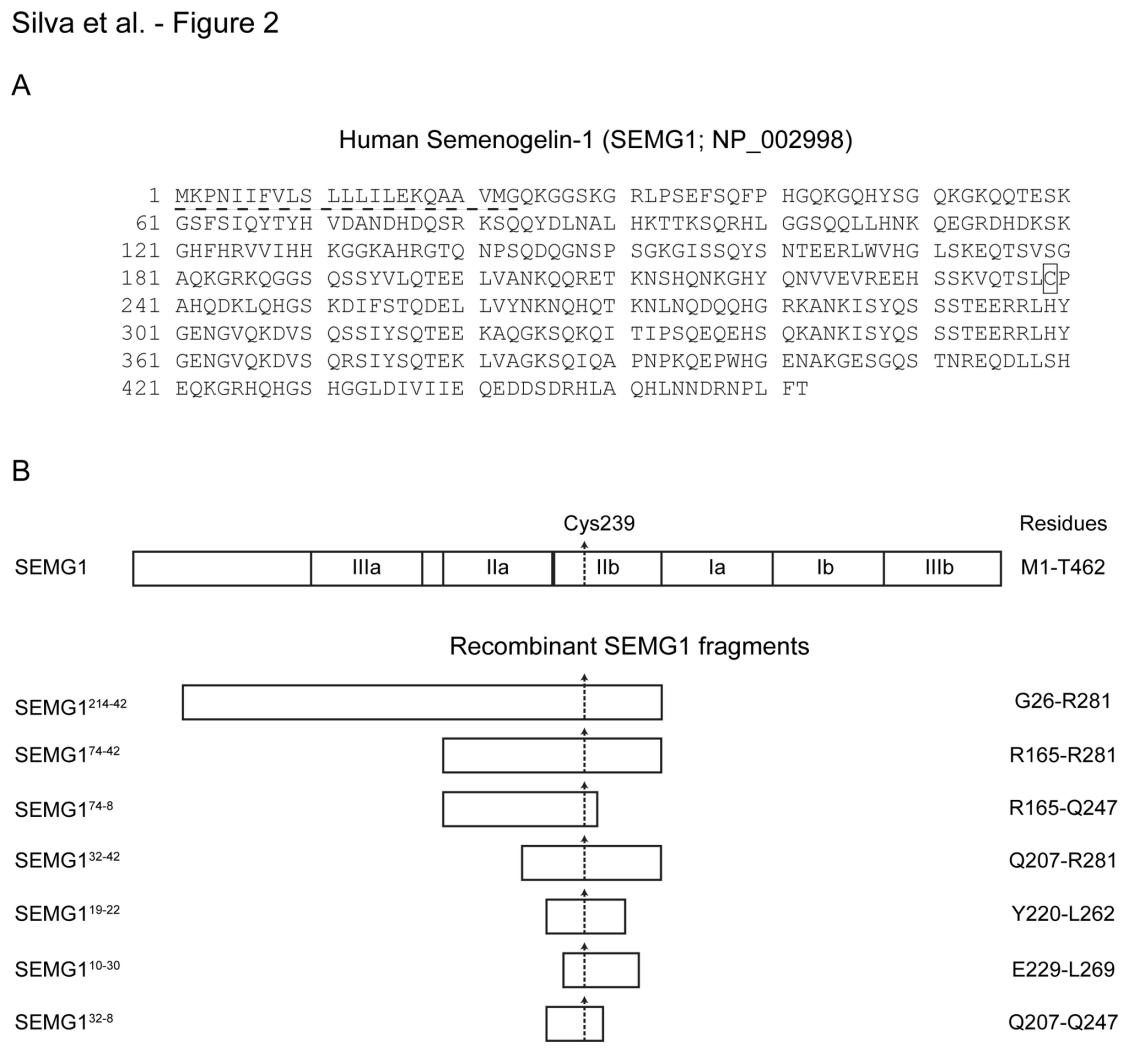
Human semenogelin I (SEMG1) sequence. **A**) SEMG1 primary sequence. Signal peptide is indicated by a dashed underline. **B**) Schematic representation of SEMG1 containing repeats Ia, Ib, IIa, IIb, IIIa, and IIIb. Recombinant SEMG1 fragments used in AlphaScreen assay and CASA experiments: G26-R281 (SEMG1^214-42^), R165-R281 (SEMG1^74-42^), R165-Q247 (SEMG1^74-8^), Q207-R281 (SEMG1^32-42^), Y220-L262 (SEMG1^19-22^), E229-L269 (SEMG1^10-30^), and Q207-Q247 (SEMG1^32-8^) are shown. Cysteine residue at position 239 is indicated by a box in (**A**) and a vertical arrow in (**B**).

Analysis of the SEMG1 sequence in fragment 74-8 (R165-Q247; Figure **2**), a fragment still retaining EPPIN binding and sperm motility inhibitory activities (see below), revealed that the Hominoidea underwent a distinct change in four amino acid residues (Trp167, His169, Ser193, and Gln215), which might be indicative of a directional selection in Hominidae (Table **4**). Additionally, Bayes Empirical Bayes (BEB) analysis [27] revealed eleven positively selected codons in this *SEMG1* sequence (Table **4**; Table **S2**); only two (Trp167, His169) were throughout the Hominoidea. Although there were no amino acid residue differences between *Homo sapiens* and all other Hominoidea, residues Asp225 and Leu234 in the *Gorilla* were distinct from other Hominidae and Hylobatidae residues, Glu225 and Val234 respectively, but identical to *Macaca mulatta* and *Papio anubis* residues (Table **4**). Residue His239 was unique in the *Gorilla* SEMG1 in comparison to SEMG1 from other Hominidae and Hylobatidae (Cys239), as well as *Macaca mulatta* and *Papio anubis* (Arg239). Residue Cys239 and His169 were reported previously to be sites under positive selection [18] and our BEB analysis confirmed that the Cys239 codon underwent positive selection (Table **4**; Table **S2**). *Homo sapiens* Cys239, which is necessary for SEMG1 binding to EPPIN [24,25], underwent a distinct change when the Hominidae and Hylobatidae Families separated from the Cercopithecidae (R239C). Consequently, based on our phylogenetic analysis, it is likely that the positive selection of eleven of *SEMG1*’s codons within the WFDC locus of the Hominoidea and the co-evolution of EPPIN’s His92 resulted in the high affinity binding of SEMG1 to EPPIN in humans.

**Table 4:** Amino acid residue changes in the SEMG1 sequence (positions 165-247).

**SEMG1**	**Species**						
Residues	**7**	**6**	**5**	**4**	**3**	**2**	**1**
(position #)			**^**				
167	R	R	***W***	***W***	***W***	***W***	W*
169	R	R	***H***	***H***	***H***	***H***	H*
176	A	A	A	***T***	A	***T***	***T***
178	A	A	A	***V***	A	***V***	***V***
184	G	G	G	G	***R***	G	G*
186	T	T	T	T	***K***	***K***	K*
192	R	R	***S***	R	***S***	***S***	S*
193	N	N	***S***	***S***	***S***	***S***	***S***
194	Y	Y	Y	Y	***H***	Y	Y*
203	A	A	A	A	***V***	A	A*
204	N	K	N	N	N	N	N
206	Q	Q	***R***	Q	Q	Q	Q
208	R	R	***G***	R	R	R	R*
211	Q	Q	Q	***K***	***K***	***K***	K*
215	R	R	***Q***	***Q***	***Q***	***Q***	***Q***
225	D	D	***E***	***E***	D	***E***	***E***
234	L	L	***V***	***V***	L	***V***	***V***
239	R	R	***C***	***C***	***H***	***C***	C*†
242	H	H	***Y***	H	H	H	H
244	H	H	***D***	***G***	***D***	***D***	***D***
245	K	K	***R***	***N***	***R***	K	K*

^ indicates the divergence of the Catarrhini into the Hominoidea (species 1-5) and Cercopithecoidea (species 6 and 7), 31.6 MYA; Changes after the split are shown in bold/italics. Residues 225, 234 and 239 in the *Gorilla* are unique to *Gorilla* within the Hominoidea. † Residue 239 is necessary for high affinity binding of SEMG1 to human EPPIN. * Indicates BEB analysis for positively selected codons (P>95%). Species: 1, *Homo sapiens*; 2, *Pan troglodytes*; 3, *Gorilla gorilla*; 4, *Pongo abelii*; 5, *Nomascus leucogenys*; 6, *Macaca mulatta*; 7, *Papio anubis*.

### Binding of SEMG1 fragments and Cys239 mutants to EPPIN

**Figure 3:**
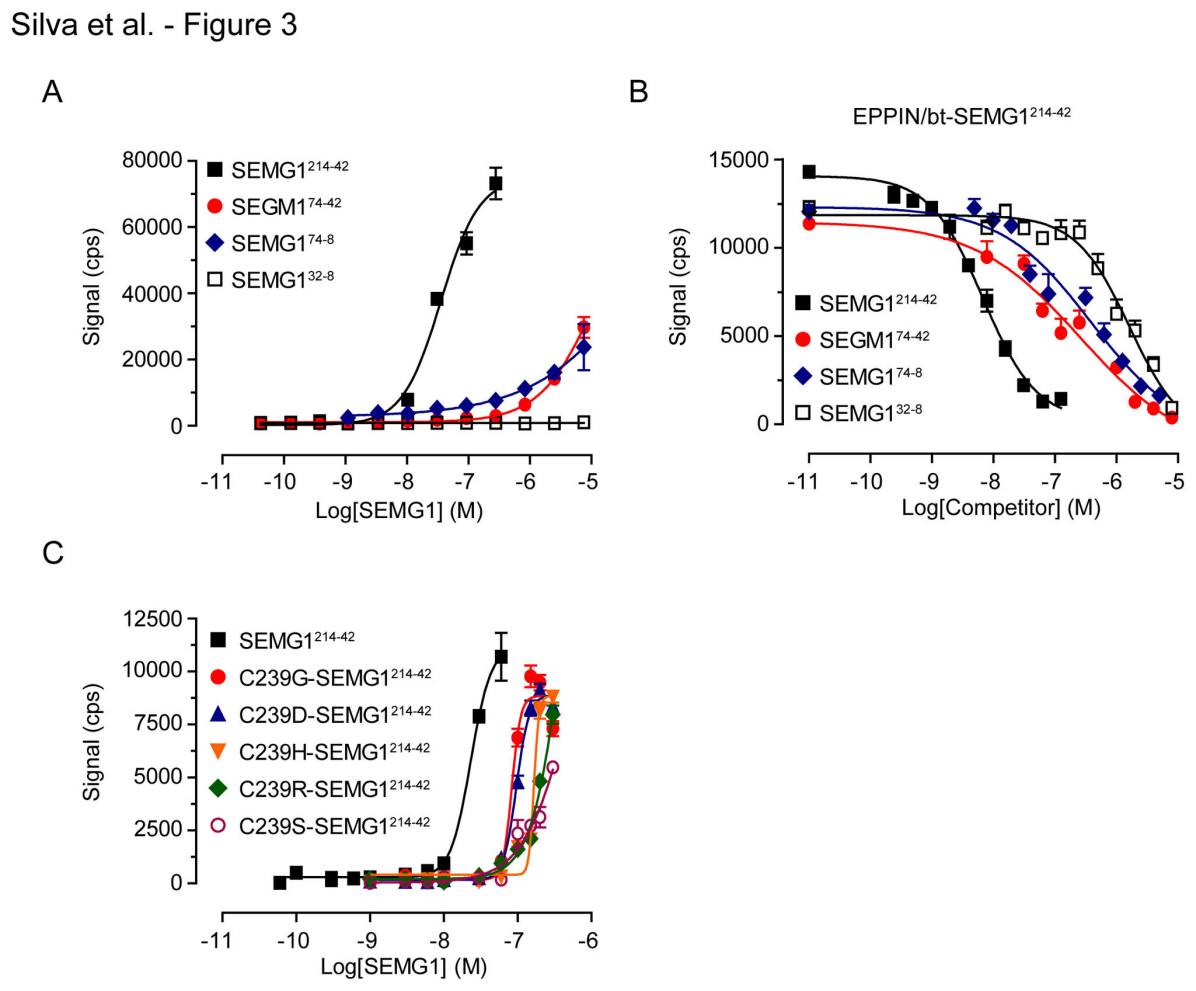
Binding of recombinant SEMG1 fragments to EPPIN in the AlphaScreen assay. **A**) Concentration-response curve for SEMG1^214-42^, SEMG1^74-42^, SEMG1^74-8^, and SEMG1^32-8^ in the presence of a constant concentration of EPPIN. **B**) Displacement of bt-SEMG1^214-42^ from its binding site on EPPIN by SEMG1 constructs. Specific signal for each data point was normalized as percentage of specific signal in the absence of SEMG1 competitors. Data are expressed as mean ± SD from two independent experiments, each performed in four replicates. **C**) Concentration-response curve for SEMG1^214-42^ (wild-type) and Cys239 mutants (C239G, C239D, C239H, C239R, C239S) in the presence of a constant concentration of EPPIN. Data points in (**A**) and (**C**) represent mean ± SD of specific signal from a representative experiment of (**A**) four (SEMG1^214-42^ and SEMG1^74-42^) and two (SEMG1^74-8^) experiments; and (**C**) three experiments, each performed in four replicates. cps = counts per second.

As we reported previously [23], the binding of human recombinant SEMG1^214-42^ fragment to EPPIN was time-dependent (Figure **S1**) and saturable (Figure **3A**). Experiments performed using increasing concentrations of SEMG1^214-42^ (0.04 - 7.5 µM) in the presence of a constant concentration of EPPIN indicated a concentration-dependent increase in the signal up to 300 nM SEMG1^214-42^ (Figure **3A** and Figure **S1**). At higher SEMG1^214-42^ concentrations, we observed a progressive drop in the signal due to oversaturation of Ni-NTA-chelate donor beads with SEMG1^214-42^ (the hook effect [28], Figure **S1**). In order to determine the smallest region within SEMG1^214-42^ critical for EPPIN binding, we cloned and expressed six SEMG1 truncated fragments lacking N-terminal and/or C-terminal sequences flanking Cys239 residue, namely SEMG1^74-42^, SEMG1^74-8^, SEMG1^32-42^, SEMG1^19-22^, SEMG1^10-30^, and SEMG1^32-8^ (Figure **2B**). We designed these truncations because they correspond to SEMG1 fragments containing the Cys239 residue generated by PSA digestion [6] and are part of the 15-kDa SEMG1 fragment protected from PSA cleavage by EPPIN [7]. Thus, it is likely they are naturally generated during the semen liquefaction. Among these fragments, only SEMG1^74-42^ and SEMG1^74-8^ bound to EPPIN, albeit at much higher concentrations (1 - 7.5 µM) in comparison to the largest fragment SEMG1^214-42^ (Figure **3A**). We observed no saturation when SEMG1^74-42^ and SEMG1^74-8^ fragments were titrated in the presence of EPPIN under similar conditions (Figure **3A**). 

To confirm these results, we performed competition experiments to investigate the capacity of SEMG1 fragments to compete with biotinylated (bt)-SEMG1^214-42^ for its binding site on EPPIN. SEMG1^214-42^ decreased bt-SEMG1^214-42^/EPPIN interaction in a concentration-dependent manner (Figure **3B**) with a calculated IC_50_ value (95% confidence interval) of 8.1 nM (6.8-9.7 nM). Both SEMG1^74-42^ and SEMG1^74-8^ inhibited the binding of bt-SEMG1^214-42^ to EPPIN at higher concentrations in comparison to SEMG1^214-42^ (Figure **3B**). The calculated IC_50_ values were 123.7 nM (92.4-165.7 nM) and 384.7 nM (104.2-1421 nM) for SEMG1^74-42^ and SEMG1^74-8^, respectively. Curiously, SEMG1^32-8^ competed with bt-SEMG1^214-42^ for binding EPPIN at even higher concentrations (Figure **3B**), with an IC_50_ of 5.1 µM (0.3-75.2 µM).

Next, we measured the binding of the SEMG1^214-42^ Cys239 point mutants to EPPIN in the AlphaScreen assay. All point mutations negatively affected SEMG1/EPPIN binding as demonstrated by right-shifted concentration-response curves (Figure **3C**) and consequently increased EC_50_ values (Table **5**) in comparison to the wild-type when SEMG1^214-42^ mutants were incubated with EPPIN under similar conditions. We observed a ~4-fold, ~8-fold and ~20-fold increase in the EC_50_ values of SEMG1/EPPIN binding when glycine/aspartic acid, histidine or serine/arginine residues replaced the Cys239 residue, respectively (Table **5**). Altogether, we confirmed previous observations that the Cys239 residue within human SEMG1 primary sequence plays a critical role in the SEMG1/EPPIN interaction. Additionally, our results indicated that sequences within SEMG1 repeats IIIa, IIa and IIb flanking Cys239 residue may provide interacting interfaces that significantly enhance SEMG1/EPPIN interaction. 

**Table 5:** EC50 values for the binding of each indicated SEMG1 construct to EPPIN by AlphaScreen assay.

**SEMG1 isoform**	**EC_50_ (95% conf. interval)**
SEMG1^214-42^	23.1 nM (21.3 - 25.0 nM)
C239G-SEMG1^214-42^	83.3 nM (76.1 - 91.3 nM)
C239D-SEMG1^214-42^	96.1 nM (91.5 - 101.0 nM)
C239H-SEMG1^214-42^	167.0 nM (160.0 - 174.0 nM)
C239R-SEMG1^214-42^	461.0 nM (97.4 - 2180 nM)
C239S-SEMG1^214-42^	564.4 nM (8.3 - 3836 nM)

EC50 calculations were performed with data showed in Figure **3C**.

### Effect of SEMG1 fragments and Cys239 mutants on sperm motility

We have previously demonstrated that the recombinant SEMG1^214-42^ fragment inhibited different sperm movement-related parameters, including motility, progressive motility and VSL (straight line velocity) of human ejaculate spermatozoa in a time- and concentration-dependent manner [24]. To determine the minimum sequence within SEMG1^214-42^ that inhibits sperm motility, we incubated spermatozoa with different recombinant SEMG1 fragments (Figure **2**) and assessed by Computer-assisted sperm analysis (CASA). Based on observations that both sperm motility and velocity are directly correlated to the fertilizing ability of human spermatozoa [29] and that SEMG1 forms a gelatinous mass after ejaculation restricting sperm movement, we developed an index (iRMI) correlating both %motility and VSL parameters, which allowed the normalization of each data set against its respective control, reducing inter-assay variation due to differences in sperm quality in different semen samples.

**Figure 4:**
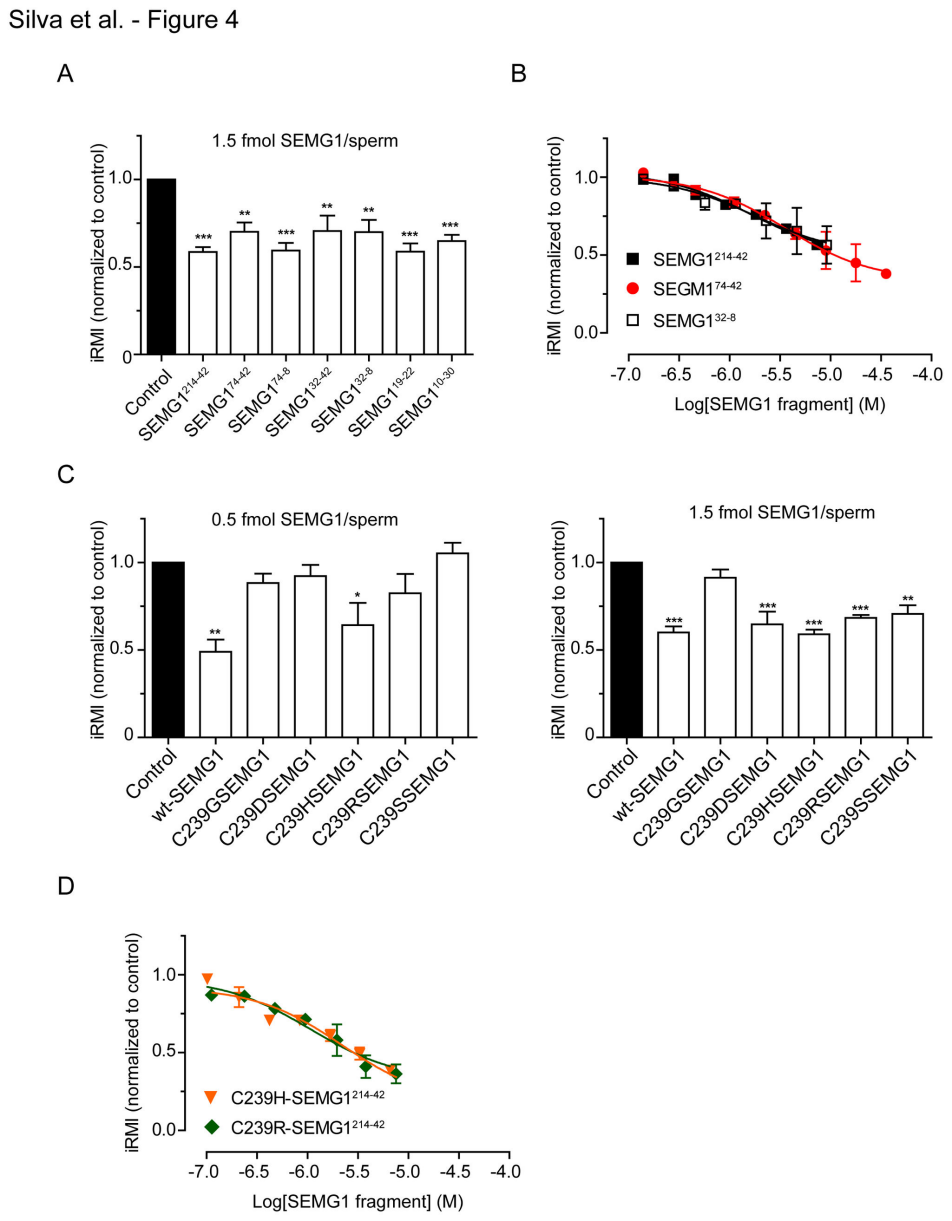
Effect of recombinant SEMG1 constructs on human sperm motility. Spermatozoa were incubated with the same concentration of recombinant SEMG1 fragments (**A**) or Cys239 mutants (**C**) and assessed by CASA. Concentration-response curves for spermatozoa incubated in the presence of increasing concentrations of SEMG1^214-42^, SEMG1^74-42^, and SEMG1^32-8^ fragments (**B**) or C239H-SEMG1^214-42^ and C239R-SEMG1^214-42^ (**D**) were obtained. Results are presented as normalized iRMI (%motility*VSL of sample/%motility*VSL of control). Control untreated experiments were performed with buffer only. Data are representative of mean ± SD of independent experiments performed with ejaculate spermatozoa from six (**A**) or three (**B**, **C** and **D**) donors. *p < 0.05, ** *p* < 0.01, and *** *p* < 0.001 when compared to control group (ANOVA followed by Bonferroni test).

As expected, SEMG1^214-42^ at a concentration of 1.5 fmol/sperm (2 µM) significantly decreased the normalized iRMI in comparison to control group (Figure **4A**). Surprisingly, all SEMG1 fragments tested significantly affected sperm motility, including those that did not bind EPPIN in the AlphaScreen assay, as demonstrated by a decrease in the normalized iRMI equivalent to that observed with SEMG1^214-42^ at the same concentration (Figure **4A**). To investigate the nature of these results, we performed concentration-response experiments in which spermatozoa were incubated with increasing concentrations of recombinant SEMG1^214-42^, SEMG1^74-42^ and SEMG1^32-8^ fragments under similar conditions and the normalized iRMI was then calculated for each concentration point (Figure **4B**). All fragments tested decreased the normalized iRMI in a concentration-dependent fashion (Figure **4B**). The calculated EC_50_ values (95% confidence interval) were 1.69 µM (0.77 - 3.7 µM), 3.56 µM (1.67 - 7.63 µM), and 1.75 µM (0.50 - 6.06 µM) for SEMG1^214-42^, SEMG1^74-42^ and SEMG1^32-8^ fragments, respectively. Based on these results, we conclude that a region within the sequence E229-Q247 of SEMG1 contains a domain responsible for the inhibition of sperm motility.

CASA experiments performed with SEMG1^214-42^ Cys239 mutants demonstrated that only C239H-SEMG1 mutant (His239 in the *Gorilla*, Table **4**) significantly decreased the normalized iRMI in a similar extent to the wild-type at a concentration of 0.5 fmol/sperm (Figure **4C** **, left panel**). When the concentration was increased to 1.5 fmol/sperm all the SEMG1 mutants, except C239G-SEMG1, significantly decreased the normalized iRMI in comparison to control (Figure **4C**, **right panel**). To confirm the concentration-dependent sperm motility inhibition of the SEMG1 Cys239 mutants, we performed concentration-response experiments using C239H-SEMG1 and C239R-SEMG1 (Figure **4D**). Both mutants decreased the normalized iRMI in a concentration-dependent manner (Figure **4D**). The calculated EC_50_ values (95% confidence interval) were 1.08 µM (0.41 - 2.84 µM) and 2.21 µM (1.14 - 4.29 µM) C239H-SEMG1 and C239R-SEMG1, respectively. 

## Discussion

Although previous reports indicate that different *WFDC* and *SEMG* genes within the *WFDC* locus are subject to rapid adaptive evolutionary changes [18,19,22,30], little is known about the molecular co-evolution of interacting proteins within this locus. The present study investigated the hypothesis that the interacting surface of SEMG1 and EPPIN co-evolved within the Hominoidea time scale, as a result of adaptive pressures applied by their roles in sperm protection and reproductive fitness. The binding of SEMG1 to EPPIN on the surface of spermatozoa is an important step for modulating sperm motility and providing antimicrobial protection upon ejaculation [5,7]. Our results indicate that distinct amino acid residues of SEMG1 (Trp167, His169, Ser193, and Gln215) and EPPIN (Lys35, Lys50, Asp51, Gln55, Lys81, Tyr89, Leu91, Met104, and Lys120) possess a remarkable deficiency of variation among hominoid primates. Using CodeML software in the PAML4 package [31] our analysis revealed that *SEMG1* codons within the fragment that binds to EPPIN underwent positive selection, confirming that there has been selective pressure on SEMG1’s EPPIN binding domain. We confirmed that SEMG1’s Cys239 residue within the SEMG1/EPPIN interacting interface is under positive selection. Although other factors such as premature stop codons and gene deletions or homogenizations may have accounted for positive selection during the evolutionary history of *SEMG1* [18], in humans Cys239’s role in increasing the binding affinity of SEMG1 for EPPIN results in a functional gain in the modulation of sperm motility in the semen coagulum at lower concentrations. In fact, our binding and functional studies in vitro demonstrate that SEMG1 truncations flanking the Cys239 residue or point mutations at this position decrease the binding of SEMG1 to EPPIN and reduce its sperm motility inhibitory effect in a concentration-dependent fashion. Although none of EPPIN’s codons underwent positive selection, which may indicate the conserved stability of *EPPIN* itself, there were two distinct changes in humans compared to all other Hominoidea; residues His92 and Asp99. Altogether, our results provide unique insights into the co-evolution of binding partners on the surface of ejaculate spermatozoa. 

Previously, we demonstrated that blocking human SEMG1’s unique cysteine residue (Cys239) either by reduction and carboxymethylation or by a point mutation to a glycine blocked its capacity to bind EPPIN and to inhibit sperm motility [24,25], indicating that this residue played a major role in SEMG1 biological activities. In an effort to further characterize SEMG1 sequences responsible for binding EPPIN and inhibiting sperm motility we tested the capacity of multiple recombinant SEMG1 fragments and Cys239 mutants to bind EPPIN and to inhibit human sperm motility. It is noteworthy that all smaller SEMG1 constructs tested correspond to SEMG1 fragments generated by PSA digestion upon ejaculation [6], making them endogenous to the liquefied semen. Our results demonstrated that the whole G26-R281 sequence in SEMG1^214-42^ was necessary for optimal SEMG1/EPPIN interaction, since this construct had the lowest EC_50_ and IC_50_ values among the SEMG1 fragments tested in concentration-response and competition experiments, respectively. 

 Interestingly, micromolar concentrations of the SEMG1^32-8^ construct were able to inhibit the interaction between EPPIN and bt-SEMG1 in competition experiments, although it did not directly bind to EPPIN in concentration-response experiments. Besides the reduced capacity of this fragment to bind EPPIN at higher concentrations, it is possible that aggregation between SEMG1^32-8^ and bt-SEMG1 may have masked the EPPIN binding site in bt-SEMG1 in our assay conditions, thereby leading to a concentration-dependent reduction in the signal. In addition, we cannot discard the possibility that the N-terminal 6X His tag present in the recombinant proteins may disturb the binding capacity of the smallest SEMG1 constructs to EPPIN due to steric/folding issues, which could lead to the absence of interaction in the direct binding assay at the concentrations tested. 

Despite the importance of the Cys239 residue to SEMG1 biological activity, our phylogenetic analysis demonstrated that this residue is not highly conserved among primates; positively charged residues histidine or arginine replace the cysteine in SEMG1 from *Gorilla gorilla* and Cercopithecoidea species *Macaca mulatta* and *Papio anubis*, respectively. Based on these observations, we decided to further investigate the physiological significance of this change to SEMG1 function by mutating Cys239 (cysteine = polar) residue in human SEMG1^214-42^ to histidine (basic), arginine (hydrophilic/basic), as well as glycine (hydrophobic), aspartic acid (negatively charged) and serine (hydrophilic) residues. Our results demonstrated that all SEMG1 mutants required much higher concentrations than the wild-type to bind to EPPIN, as indicated by higher EC_50_ values in the concentration-response experiments. An interesting observation was the fact that the C239H-SEMG1 mutant possessed a ~4-fold higher EPPIN binding activity than the C239R-SEMG1 mutant based on EC_50_ values, suggesting that the size of the side-chain of the residue at this position may hamper SEMG1/EPPIN interaction by inducing steric effects. Taken together, our experimental data support the observation that human SEMG1’s Cys239 residue is a hot site subject to positive selection, probably because of its major role in increasing the affinity of SEMG1 for EPPIN on the sperm surface, which can result in an effective gain of function by allowing an effective binding at lower concentrations. Our results further indicate that the sequence G26-E164, which is part of the repeat IIIa, provide a docking surface that is critical for the SEMG1-EPPIN interaction in addition to the Cys239 residue, leading us to hypothesize that SEMG1 has multiple EPPIN interaction sites within repeats IIIa and IIb of the molecule. This hypothesis is further supported by the observation that residues Cys102, Tyr107, and Phe117 in EPPIN’s SEMG1 binding pocket are independently important for SEMG1 interaction [23].

We and others have demonstrated by computer analyses of sperm motility that both native and recombinant SEMG1 decreased motility parameters, such as percentage motility and straight line velocity of human ejaculate spermatozoa in a concentration- and time-dependent fashion [15,16,24,32]. Recent studies from our laboratory have begun to unravel the basic mechanisms underlying the SEMG1-mediated inhibition of sperm motility triggered by its interaction with EPPIN on the sperm surface, namely the decrease of intracellular pH leading to a drop in intracellular calcium levels by blocking extracellular calcium uptake [15,16]. These observations demonstrated that the effects of SEMG1 on sperm motility are finely regulated by a cascade of molecular events rather than by simply trapping spermatozoa by physical constraint in the semen coagulum. In our study, an intriguing observation was the fact that all smaller SEMG1 constructs tested inhibited sperm motility to a similar extent as SEMG1^214-42^, including those that did not bind EPPIN in the AlphaScreen assay. Differences in the nature of these assays may explain our results: while the AlphaScreen assay measures the interaction between EPPIN and SEMG1 molecules in vitro with high sensitivity (nanomolar range), CASA analysis reflects a biological effect in a live cell, namely loss of sperm motility, which is dependent on a cascade of molecular events initiated by the same interaction, such as changes in intracellular pH and extracellular calcium uptake [15,16]. It is possible that the saturation of EPPIN binding sites with SEMG1 on the surface of spermatozoa, which would induce the formation of large macromolecular complexes, may be required for their immobilization.

It has been widely recognized that genes with roles in innate immunity and reproduction are evolving rapidly by positive Darwinian selection, particularly those associated with the male reproductive tract [33-35]. Consistently, the evolution of different sperm- and semen-associated proteins, such as *SEMG* and *WFDC* genes in the primate lineage, is rapid and driven by positive selective forces, especially in relation to sexual function and sperm competition [19]. In fact, the nearly significant heterogeneity between *Gorilla* and *Homo sapiens SEMG1* further suggests an ongoing divergent evolutionary trend after speciation. Our results suggest that the adaptive molecular coevolution of the interacting surface between SEMG1 and EPPIN may have rapidly changed their biochemical properties resulting in an increase in their binding affinity and a gain of function for both proteins at the interface of innate immunity and reproduction. 

## Materials and Methods

### Phylogenetic analysis

We aligned nucleotides sequences of *EPPIN* and *SEMG1* from *Homo sapiens*, *Pan troglodytes*, *Gorilla gorilla gorilla*, *Pongo abelii*, *Nomascus leucogenys*, *Macaca mulatta*, *Papio anubis*, and *Saimiri boliviensis boliviensis* (GenBank accession numbers in Table **6**) with ClustalW. We used MEGA5 molecular evolutionary genetic analysis software package (version 5.1) [36] to obtain phylogenetic trees using the Maximum Likelihood method based on the Tamura-Nei model [37] and to perform the test of homogeneity of substitution patterns between sequences for *EPPIN* and *SEMG1* using the neighbor-joining method [38]. Codon positions included were 1st+2nd+3rd. All positions containing gaps and missing data were eliminated. For technical details of these tests, please refer to their original descriptions [36-38]. We chose the phylogenetic trees with the highest log likelihood (*EPPIN*) -926.2396 or (*SEMG1*) -2108.0861 and used them for further analysis. We investigated evidence of positive selection codon-by-codon in the primate phylogenetic tree of *EPPIN* and *SEMG1* using the CodeML software in the PAML 4 package [27,31]. In this case, we compared the likelihood of a nearly-neutral model M1a with that of a selection model M2a. Next, we compared the likelihood of a neutral model M7 with that of a selection model M8. In both cases we used a Likelihood Ratio Test (LRT) to determine if the selection model was a better fit to the data than the neutral model by comparing the (-2[Log(neutral) – Log(positive)]) value between the two models with the χ^2^-distribution [18,27]. When the calculations suggested positive selection, we used the BEB method to calculate the probability that each codon experienced positive selection under models M2a and M8 [27]. 

**Table 6:** List of species and sequences used in the phylogenetic analysis of EPPIN and SEMG1.

	**GenBank Accession Number**	
**Species**	***EPPIN***	***SEMG1***
*Homo sapiens*	NM_020398.3	NM_003007.3
*Pan troglodytes*	XM_003316966.1	NM_001110538.1
*Gorilla gorilla gorilla*	XM_004062257.1	XM_004062223.1
*Pongo abelii*	XM_002830353.1	XM_002830350.1
*Nomascus leucogenys*	XM_003253642.1	gi|328833490:8997382-9005052
*Macaca mulatta*	NM_001032841.1	XM_002798120.1
*Papio anubis*	XM_003904690.1	NM_001169022.1
*Saimiri boliviensis boliviensis*	XM_003936444.1	gb|DP000047.1|:154056-155465

### Production and Expression of Recombinant Proteins

Human recombinant SEMG1 (SEMG1^214-42^, wild-type, residues G26-R281, Figure **2**) and EPPIN (residues P22-P133) were previously cloned into pET100/D-TOPO vector (Invitrogen, Carlsbad, CA) [23]. Additionally, C-terminal and N-terminal truncated fragments flanking the residue Cys239 from the SEMG1 primary sequence, corresponding to residues R165-R281 (SEMG1^74-42^), R165-Q247 (SEMG1^74-8^), Q207-R281 (SEMG1^32-42^), Y220-L262 (SEMG1^19-22^), E229-L269 (SEMG1^10-30^), and Q207-Q247 (SEMG1^32-8^) (Figure **2B**) were produced and cloned into the same vector. All constructs were verified by DNA sequencing. Recombinant proteins were expressed either in BL21 Star^TM^ (DE3) One Shot^®^ *E. coli* (Invitrogen; SEMG1 constructs) or *E. coli* Rosetta-Gami 2^TM^ (DE3) (Novagen, Madison, WI; EPPIN construct) and purified under denaturing conditions using nickel-nitrilotriacetic acid agarose (Ni-NTA) beads (Qiagen, Valencia, CA) as described previously [23]. 

Removal of N-terminal 6X His-tag from recombinant EPPIN was performed using recombinant enterokinase cleavage/capture kit (Novagen), according to the manufacturer’s instructions. For removal of undigested 6X His-tag-EPPIN, the cleavage reaction was incubated with Ni-NTA beads at room temperature for 30 min with gentle mixing and tag-free EPPIN was then eluted in PBS buffer containing 1 M urea. Recombinant SEMG1^214-42^ was biotinylated as described previously [23].

### In Vitro Site-Directed Mutagenesis

Site-directed mutagenesis of wild-type SEMG1^214-42^ of residue Cys239 to glycine (C239G-SEMG1), aspartic acid (C239D-SEMG1), histidine (C239H-SEMG1), serine (C239S-SEMG1), or arginine (C239R-SEMG1) was performed using the Gene Tailor site-directed mutagenesis system (Invitrogen), according to the manufacturer’s instructions. Mutagenesis products were transformed into DH5a-T^1R^ *E. coli* and positive clones selected, followed by DNA sequencing to confirm the mutation. Recombinant SEMG1 mutants were expressed and purified as described above.

### AlphaScreen^®^ Assay

The AlphaScreen^®^ assay was carried as previously described [23], with the following modifications. Briefly, recombinant EPPIN was pre-incubated with anti-EPPIN Q20E antibody and Protein A acceptor beads for 30 minutes. In parallel, increasing concentrations of each recombinant SEMG1 construct was incubated with Ni-NTA-chelate donor beads under the same conditions. Equal volumes of each EPPIN/Q20E/Protein A acceptor beads and SEMG1/Ni-NTA-chelate donor beads were pipetted into in white opaque 384-well microplates (OptiPlate-384; PerkinElmer, Waltham, MA) in a final volume of 30 µl. Plates were covered with top seal and transferred to a Synergy 2 Multiplatform automated plate reader (Biotek, Winooski, VT). After shaking for 2 min, plates were read every 2 h during 16 h: excitation using a 680/30 filter, emission using a 570/100 filter and data acquired using Gen5 software (Biotek). A total of 9 time points were generated during each experiment. Each set of samples was pipetted in 4 replicates. The final concentration of assay components was 29 nM EPPIN, 2 nM Q20E antibody, 0.04-7.5 µM SEMG1, and 10 µg/ml beads. Negative controls were performed under the same conditions in the absence of EPPIN or SEMG1 and in the presence of beads only. A specific signal for each time point was calculated by subtracting the background signal (obtained in the absence of SEMG1) from its respective total signal. EC50 values for the binding of EPPIN to each SEMG1 construct were calculated by non-linear regression curve fitting using the specific signal obtained after 16 h of incubation. 

Competition experiments using AlphaScreen assay platform were performed as previously described [23]. Briefly, EPPIN (10 nM) and biotinylated (bt-)SEMG1^214-42^ (1 nM) were incubated in the presence of increasing concentrations of SEMG1^214-42^, SEMG1^74-42^, SEMG1^74-8^, or SEMG1^32-8^ (10 pM – 8 µM) in a 30 µl reaction. The bead concentration was 15 µg/ml. The specific signal for each competitor concentration point was calculated as described above and the IC_50_ values were calculated by nonlinear regression curve fitting.

### Preparation of spermatozoa and analysis of sperm motility

Human semen samples were obtained from the Department of Obstetrics and Gynecology, University of North Carolina Memorial Hospital, Chapel Hill, NC. This study was approved by the Committee on the Protection of the Rights of Human Subjects at the University of North Carolina, School of Medicine Chapel Hill, NC. The Institutional Review Board determined that this study did not constitute human subjects research as defined under U.S. federal regulations [45 CFR 46.102 (d or f) and 21 CFR 56.102(c)(e)(l)] and the need for informed consent was waived. All samples were de-identified before further processing. Semen samples were allowed to liquefy for 30 min and subjected to standard semen analysis to determine acceptability and either used fresh or stored in liquid nitrogen. An isolate gradient (Irving Scientific, Irving, CA, cat. #99264) was used to prepare spermatozoa for further analysis [15]. 

Aliquots of sperm suspension (1500 cells/µl) were incubated with increasing concentrations of each SEMG1 construct (0 - 35.6 µM; diluted in modified M16 medium no phenol red (Millipore, Billerica, MA, cat. #MR/010/D) supplemented with 50 µM ZnCl_2_ in 12- X 75-mm glass tubes at 37°C and 5% CO2 for 1-2 h. We repeated each experiment with spermatozoa from at least three different ejaculates and evaluated at least 100 cells (range 100-1000 cells) from each data point. 

The analysis of sperm motility after incubation with SEMG1 constructs was carried out using computer-assisted sperm analysis (CASA; Ceros version 14.8 software, Hamilton-Thorne), as previously described [24]. In each case, the percentage sperm motility (%motility) and straight-line velocity (VSL; the average velocity measured in a straight line from the beginning to the end of track in µm/sec) were used to calculate the index of relative motility inhibition (iRMI) as: %motility*VSL. Normalized iRMI was calculated by dividing the iRMI of each experimental condition by its respective control (absence of SEMG1).

### Statistical analysis

Results from AlphaScreen assays and CASA experiments were expressed as mean ± standard deviation (SD) or standard error of the mean (SEM) from the indicated number of independent experiments. For statistical significance, one-way analysis of variance (ANOVA) followed by the Bonferroni test was performed using GraphPad Prism 5.0 software. *p* < 0.05 was considered statistically significant.

## Supporting Information

Table S1 **Likelihood ratio test between different evolutionary models for EPPIN (A) and *SEMG1* (B).**
(PDF)

Table S2 **Log-likelihood values and parameter estimates for *SEMG1*.**
(PDF)

Figure S1 **Characterization of the interaction between EPPIN and his-SEMG1 (SEMG1^214-42^ fragment) in the AlphaScreen Assay.** A) Time-course experiment showing the interaction between SEMG1^214-42^ and EPPIN. Background signal was detected when beads were incubated in the absence of SEMG1^214-42^. B) Concentration-response curve for SEMG1^214-42^ in the presence of a constant concentration of EPPIN. A reduction in the signal (hook effect, arrow) was observed with his-SEMG1 concentrations higher than 300 nM. Negative control was performed in the absence of EPPIN. Specific signal for each data point was determined by subtracting the background signal from total signal. Data points represent mean ± SD of specific signal from a representative experiment of four experiments, each performed in four replicates. cps = counts per second.
(PDF)
