# Supplementary material for: Interacting Proteins on Human Spermatozoa: Adaptive Evolution of the Binding of Semenogelin I to EPPIN
Source: PLoS One. 2013 Dec 2;8(12):e82014. doi: 10.1371/journal.pone.0082014 (PMC3846889; doi:10.1371/journal.pone.0082014)
Supplement: Table S1 — Likelihood ratio test between different evolutionary models for EPPIN (A) and SEMG1 (B). (PDF) [file pone.0082014.s001.pdf]

Supplementary Information

"Interacting Proteins on Human Spermatozoa: Adaptive Evolution of the Binding of Semenogelin I to EPPIN"

Erick J.R. Silva, Katherine G. Hamil, and Michael G. O'Rand

Supplementary Table S1. Likelihood ratio test between different evolutionary models for *EPPIN* (A) and *SEMG1* (B).

Evolutionary model	Degrees of Freedom	LRT	P-value
**(A) *EPPIN***			
M1a (null) vs M2a (positive)	2	0.2600	0.8781
M7 (null) vs M8 (positive)	2	0.3446	0.8418
**(B) *SEMG1***			
M1a (null) vs M2a (positive)	2	14.0926	0.0009*
M7 (null) vs M8 (positive)	2	14.0950	0.0009*

Degree of freedom indicates the difference in the number of parameters between models; LRT (Log-likelihood ratio test) was calculated using the formula (-2[Log(null) – Log(positive)]) to compare the better fit model with the χ^2-distribution; P-value is the probability that two models should differ in log likelihood as much as that observed, given the degree of freedom. *P<0.05 was considered significant.
