# Supplementary material for: Interacting Proteins on Human Spermatozoa: Adaptive Evolution of the Binding of Semenogelin I to EPPIN
Source: PLoS One. 2013 Dec 2;8(12):e82014. doi: 10.1371/journal.pone.0082014 (PMC3846889; doi:10.1371/journal.pone.0082014)
Supplement: Table S2 — Log-likelihood values and parameter estimates for SEMG1. (PDF) [file pone.0082014.s002.pdf]

Supplementary Information

"Interacting Proteins on Human Spermatozoa: Adaptive Evolution of the Binding of Semenogelin I to EPPIN"

Erick J.R. Silva, Katherine G. Hamil, and Michael G. O'Rand

Supplementary Table S2. Log-likelihood values and parameter estimates for *SEMG1*.

**Evolutionary model**	***p***	**Log likelihood**	**Estimates of parameters**	**Positive selected sites by BEB method (P>95%)**
M0 (one-ratio)	1	-2183.66	ω=1.67	N/A
M1a (nearly-neutral)	2	-2188.02	p_0=0.005(p_1=0.995), ω_0=0.000(ω_1=1)	N/A
M2a (selection)	4	-2180.97	p_0=0.413, p_1=0.000(p=0.587), ω_0=0.595(ω_1 = 1), ω_2=2.534	117D
M7 (beta)	2	-2188.02	p=1.391, q=0.005	N/A
M8 (beta&ω)	4	-2180.97	p_0=0.413(p_1=0.587), $p_=$99.000, q=66.515, ω=2.53414	73A, 97Q, 117D, 125R, **167W, 169H, 184G, 186K, 192S, 194Y, 203A, 208R, 211K, 239C, 245K**

p indicates the number of parameters in the ω distribution; Data were based on a non-rooted tree; single letter amino acid designation. There were a total of 256 positions in the dataset. Residues within SEMG1 fragment 74-8 (R165-Q247) are shown in bold.
