# Supplementary material for: Interacting Proteins on Human Spermatozoa: Adaptive Evolution of the Binding of Semenogelin I to EPPIN
Source: PLoS One. 2013 Dec 2;8(12):e82014. doi: 10.1371/journal.pone.0082014 (PMC3846889; doi:10.1371/journal.pone.0082014)
Supplement: Figure S1 — Characterization of the interaction between EPPIN and his-SEMG1 (SEMG1214-42 fragment) in the AlphaScreen Assay. A) Time-course experiment showing the interaction between SEMG1214-42 and EPPIN. Background signal was detected when beads were incubated in the absence of SEMG1214-42. B) Concentration-response curve for SEMG1214-42 in the presence of a constant concentration of EPPIN. A reduction in the signal (hook effect, arrow) was observed with his-SEMG1 concentrations higher than 300 nM. Negative control was performed in the absence of EPPIN. Specific signal for each data point was determined by subtracting the background signal from total signal. Data points represent mean ± SD of specific signal from a representative experiment of four experiments, each performed in four replicates. cps = counts per second. (PDF) [file pone.0082014.s003.pdf]

Supplementary Information

"Interacting Proteins on Human Spermatozoa: Adaptive Evolution of the Binding of Semenogelin I to EPPIN"

Erick J.R. Silva, Katherine G. Hamil, and Michael G. O'Rand

Silva et al. - Supplementary Figure S1

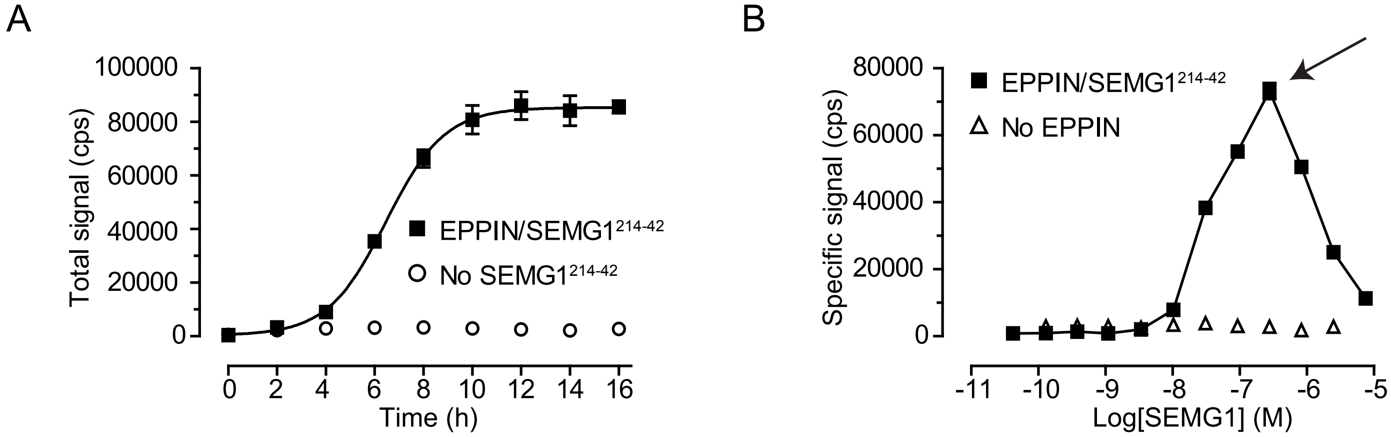

Supplementary Figure S1. Characterization of the interaction between EPPIN and his-SEMG1 ($SEMG1^{214-42}$ fragment) by the AlphaScreen Assay. **A**) Time-course experiment showing the interaction between $SEMG1^{214-42}$ and EPPIN. Background signal was detected when beads were incubated in the absence of $SEMG1^{214-42}$. **B**) Concentration-response curve for $SEMG1^{214-42}$ in the presence of a constant concentration of EPPIN. A reduction in the signal (hook effect, arrow) was observed with his-SEMG1 concentrations higher than 300 nM. Negative control was performed in the absence of EPPIN.Specific signal for each data point was determined by subtracting the background signal from total signal. Data points represent mean ± SD of specific signal from a representative experiment of four experiments, each performed in four replicates. cps = counts per second.
